# Investigation of Anti-Tumor Effects of an MLK1 Inhibitor in Prostate and Pancreatic Cancers

**DOI:** 10.3390/biology10080742

**Published:** 2021-08-02

**Authors:** Yu-Ching Fan, Kai-Cheng Hsu, Tony-Eight Lin, Dietmar Zechner, Sung-Po Hsu, Yuan-Chin Tsai

**Affiliations:** 1 PhD Program for Cancer Molecular Biology and Drug Discovery, College of Medical Science and Technology, Taipei Medical University and Academia Sinica, Taipei 11031, Taiwan; d621109001@tmu.edu.tw (Y.-C.F.); piki@tmu.edu.tw (K.-C.H.); 2 Graduate Institute of Cancer Biology and Drug Discovery, College of Medical Science and Technology, Taipei Medical University, Taipei 11031, Taiwan; tonyelin@tmu.edu.tw; 3 Cancer Center, Wan Fang Hospital, Taipei Medical University, Taipei 11600, Taiwan; 4 TMU Research Center of Cancer Translational Medicine Taipei, Taipei 11031, Taiwan; 5 Master Program for Cancer Molecular Biology and Drug Discovery, College of Medical Science and Technology, Taipei Medical University, Taipei 11031, Taiwan; 6 Institute for Experimental Surgery, Rostock University Medical Center, Schillingallee 69a, 18059 Rostock, Germany; dietmar.zechner@uni-rostock.de; 7 Department of Physiology, School of Medicine, College of Medicine, Taipei Medical University, Taipei 11031, Taiwan; sphsu@tmu.edu.tw

**Keywords:** MLK1, androgen receptor, prostate cancer, pancreatic cancer

## Abstract

**Simple Summary:**

Both prostate and pancreatic cancers are ranked in the top five leading causes of cancer death in American. In prostate cancer, the mainstay of therapeutic approaches is inhibition of the androgen receptor; however, resistance occurs within two years. In pancreatic cancer, there is no targeted therapy available, and patients have the worst survival rate compared to all other types of cancer. We identified a novel MLK1 inhibitor (NSC14465) and demonstrated anti-tumor ability in both prostate and pancreatic cancers.

**Abstract:**

It was shown that mixed lineage kinase 1 (MLK1) regulates pancreatic cancer growth; however, its role in prostate cancer remains unclear. We showed that MLK1 is a tumor marker in prostate cancer by analyzing clinical gene expression data and identified a novel MLK1 inhibitor (NSC14465) from the compound library of the National Cancer Institute (NCI) using a MLK1 protein structure. The inhibitory effects of MLK1 were validated by an in vitro kinase assay and by monitoring phosphorylation signaling, and the anti-proliferation function was shown in several prostate and pancreatic cancer cell lines. We also demonstrated anti-tumor ability and prevention of cancer-related weight loss in a syngeneic orthotopic mouse model of pancreatic cancer that mimicked the tumor growth environment in the pancreas. Our results demonstrate that the MLK1 inhibitor is an anti-tumor agent for malignant prostate and pancreatic cancers.

## 1. Introduction

According to the American Cancer Society, prostate cancer is the second leading cause of cancer death in American men and pancreatic cancer was the fourth in both men and women in 2021 [1]. In prostate cancers, castration-resistant prostate cancer (CRPC) occurs within a few years of androgen-deprivation therapy (ADT), and a key driver of the malignant progression is the androgen receptor (AR) [2]. Although therapeutics targeting AR activities have improved survival rates, none of these are curable, and resistance emerges again [3]. Pancreatic cancer has the worst survival rate of all types of cancers, with five-year survival rates of 2.5–8.2% [4]. Currently, using a surgical approach in combination with adjuvant chemotherapies is the standard therapeutic approach [5]; however, there are still unmet medical needs for treating pancreatic cancer.

The mitogen-activated protein kinase (MAPK) pathway consists of three sequential layers of kinase activity that deliver signals by protein phosphorylation in a sequential order: MAPK kinase kinases (MAP3Ks)-MAPK kinases (MAP2Ks, e.g., MEK1)-MAPKs (e.g., ERK1/2) [6]. The mixed lineage kinase 1 (MLK1) belongs to the MLK family, a subgroup of MAP3Ks, and plays a critical role in regulating pancreatic cancer, in that MLK1 promoted proliferation and inhibited apoptosis partly via activation of MEK-ERK signaling [7]. At first, it was believed that the MLK family cannot activate MEK-ERK signaling, the mitogenic pathway for cell proliferation, which is only regulated by another subgroup of MAP3Ks, RAF proto-oncogene kinases (RAFs) [8]; however, it was later demonstrated that all the members of the MLK family (MLK1-MLK4) can activate the MEK-ERK pathway and lead to anti-cancer drug resistance in cancer patients undergoing treatments with RAF inhibitors [9]. Furthermore, in addition to overexpression, gain-of-function mutations of the MLK1 were frequently found in the melanoma, contributing to its role in cancer biology [9].

Although the MLK1 is involved in the above mentioned tumors, its role in prostate cancer remains unclear. In this study, we showed that MLK1 can serve as a tumor marker in prostate cancer and aimed to utilize MLK1 as a therapeutic target. We identified a novel MLK1 inhibitor (NSC14465) from the NCI compound library and demonstrated its anti-tumor functions in prostate and pancreatic cancer cells. Our results support that MLK1 can be a therapeutic target in treating malignant prostate and pancreatic cancers.

## 2. Materials and Methods

### 2.1. Cell Lines

The mouse cell lines, tumorigenic TRAMP-C1, and human prostate cancer cell lines (C4-2, DU145, PC3, and LNCaP) were purchased from the American Type Culture Collection (ATCC; Manassas, VA, USA). All cells were maintained in the culture medium suggested by the ATCC. The mouse pancreatic cell lines (6606PDA, 6606I, and 7265PDA) were kindly provided by Dr. David Tuveson (Cambridge University) via a material transfer agreement. The three pancreatic cancer cell lines were maintained in Dulbecco’s modified Eagle medium/F12 (Invitrogen, Carlsbad, CA, USA) with 10% fetal calf serum, according to established procedures [10]. In experiments with an AR inhibitor, MDV3100 (Selleckchem, Houston, TX, USA), cells were incubated with 10 µM MDV3100 for 24 h in 10% serum-containing RPMI 1640 medium.

### 2.2. Structure-Based Virtual Screening

Compounds from the National Cancer Institute (NCI) compound database were selected for in silico screening. The compound library was prepared by removing molecules that contained pan-assay interference compound (PAINS) structures [11] or violated Lipinski’s rule of five [12]. The remaining compounds were filtered using the HTS module in Pipeline Pilot [13], which removes molecules that are poor candidates for high-throughput screening. The prepared compound library was then docked using FlexX [14]. The MLK1 structure (PDB ID: 3DTC) was obtained from the Protein Data Bank [15]. The co-crystal ligand was selected as the reference ligand to define the binding site. Docked compounds were ranked according to their docking score. The top-ranked 300 compounds were selected for visual inspection. In addition, the docked compounds were filtered based on the presence of a hydrogen bond to hinge residues [16]. Potential inhibitors were then requested based on availability for further testing.

### 2.3. In Vitro Kinase Assay

The inhibitory effects of the selected compounds were assessed using a Thermo Fischer Scientific Z’LYTE activity assay (Thermo Fisher Scientific, Waltham, MA, USA). In brief, the assay utilizes fluorescence resonance energy transfer (FRET) to evaluate MLK1 activity. The test compound was co-incubated with a fluorescein-labelled substrate, ATP, and kinase in a buffer to induce a reaction for 1 h. A stop solution (EDTA) was added, and the results were determined using a fluorescence reader. The data shown are the average of two replicates. The kinase selectivity of NSC14465 was obtained using the Thermo Fisher SelectScreen service (Thermo Fisher Scientific, Waltham, MA, USA). Protocols and assay conditions can be found online (http://www.thermofisher.com/selectscreen, accessed on 2 June 2021).

### 2.4. Transwell and Wound-Healing Assays

A human monocyte cell line, THP-1, was placed on the upper layer of cell culture inserts with a permeable membrane (Corning, Glendale, AZ, USA) then incubated in culture media containing NSC14465 and a chemokine, mouse CCL2 recombinant protein (R&D systems, Minneapolis, MN, USA), for 3 h. Two prostate cancer lines (DU145 and PC3) and a mouse pancreatic cancer line, 6606PDA, were also used in a transwell assay for 36 h. The number of migrated cells was counted by Image J software. For a wound-healing assay, TRAMP-C1, DU145, PC3, and 6606PDA cells were seeded into 12-well plates and incubated for 24–48 h. When cells were confluent on the surface of the plate, the assay was performed by using a 200 μL pipette tip to generate a scratch in each well. Cells were incubated in culture media containing different concentrations of NSC14465 for 12 h. Images were collected at the end of the experiment.

### 2.5. Proliferation and Colony-Formation Assays

To perform the proliferation assay, different types of cells (LNCaP, C4-2, DU145, and PC3) were seeded in 96-well plates for 24 h followed by enzalutamide/MDV3100 (Selleckchem, Houston, TX, USA) or NSC14465 treatment for 3 days. A CCK-8 solution (Cell Counting Kit-8, cat no.: CK04-05. Dojindo molecular technologies Inc., Kumamoto, Japan) was applied to each well and incubated for 1 h. The measurement was made by reading the absorbance at 450 nm on an Epoch Microplate Spectrophotometer (BioTek Instruments, Winooski, VT, USA).

In a colony-formation assay, cells (1000 per well) were seeded for 24 h followed by NSC14465 for 10 days. Colonies were stained with crystal violet (Sigma-Aldrich, St. Louis, MO, USA) and photographed.

### 2.6. Animals and the Syngeneic Orthotopic Mouse Model of Pancreatic Cancer

Animal experiments were performed in accordance with a protocol approved by the Taipei Medical University Animal Care and Use Committee (number: LAC-2021-0052, Taipei, Taiwan). An established procedure to establish the syngeneic mode was followed [10]; in brief, 1 × 10^5^ 6606PDA cells were mixed with Matrigel (Corning, NY, USA) and orthotopically injected into the pancreas of 8-week-old male C57BL/6 mice (NLAC, Taipei, Taiwan). From three days after tumor cell injection, 30 μg NSC14465 dissolved in 200 μL phosphate buffered saline (PBS) was administered i.p. into mice (~1.2 mg NSC14465/kg) three times a week. Mice treated with 1% DMSO in 200 μL PBS (~80 μL DMSO/kg) served as the reference control.

### 2.7. Western Blot Analysis

Cell lysates were processed with 6× Laemmli sample buffer, followed by electrophoresis. Samples were transferred to polyvinylidene difluoride membranes and blocked with 5% milk in TBST (Sigma-Aldrich, St. Louis, MO, USA). Western blotting was performed with the following antibodies at 4 °C overnight: anti-pMAP3K9 (T312) (cat. no.: BS-6779R, Thermo Fisher Scientific, Waltham, MA, USA), anti-MAP3K9 (cat. no.: ab228752, abcam, Cambridge, UK), anti-p70 S6K, anti-p-Akt (S473), anti-Akt, anti-pMEK1/2(S217/221) (cat. no.: 9204S, 9271S, 9272, 9121S; Cell Signaling Technology, Inc., Danvers, MA, USA), and anti-GAPDH (cat. no.: GTX100118, GeneTex, Irvine, CA, USA). Membranes were washed with TBST twice and incubated with a horseradish peroxidase (HRP)-antibody at room temperature for 1 h. After incubation with an HRP substrate (WesternBright ECL HRP Substrate, Advansta, San Jose, CA, USA), images were taken with an Amersham^TM^ Imager 600 (GE Healthcare, Chicago, IL, USA).

### 2.8. Bioinformatics and Statistical Analyses

Several datasets (Wallace [17], Grasso [18], Arredouani [19], and Tomlins [20]) were used to compare the MLK mRNA expression in normal and tumor tissues using the Oncomine [21] (http://www.oncomine.org/, accessed on 2 June 2021) and Gene Expression Omnibus (GEO) databases (http://ncbi.nlm.nih.gov/geo/, accessed on 2 June 2021). Gene Expression Profiling Interactive Analysis (GEPIA2) [22] and cBioPortal [23] were used to analyze the correlation of mRNA expression. The Human Protein Atlas [24] (http://www.proteinatlas.org/, accessed on 2 June 2021) was used to analyze the overall survival in the prostate cancer patients. All data are presented as the mean ± standard deviation (SD). Differences between individual groups were analyzed by Student’s *t*-test.

## 3. Results

### 3.1. Association of Increased MLK1 Messenger Ribonucleic Acid (mRNA) with Tumors and Poor Survival Rate in Clinical Datasets of Prostate Cancers

**Figure 1:**
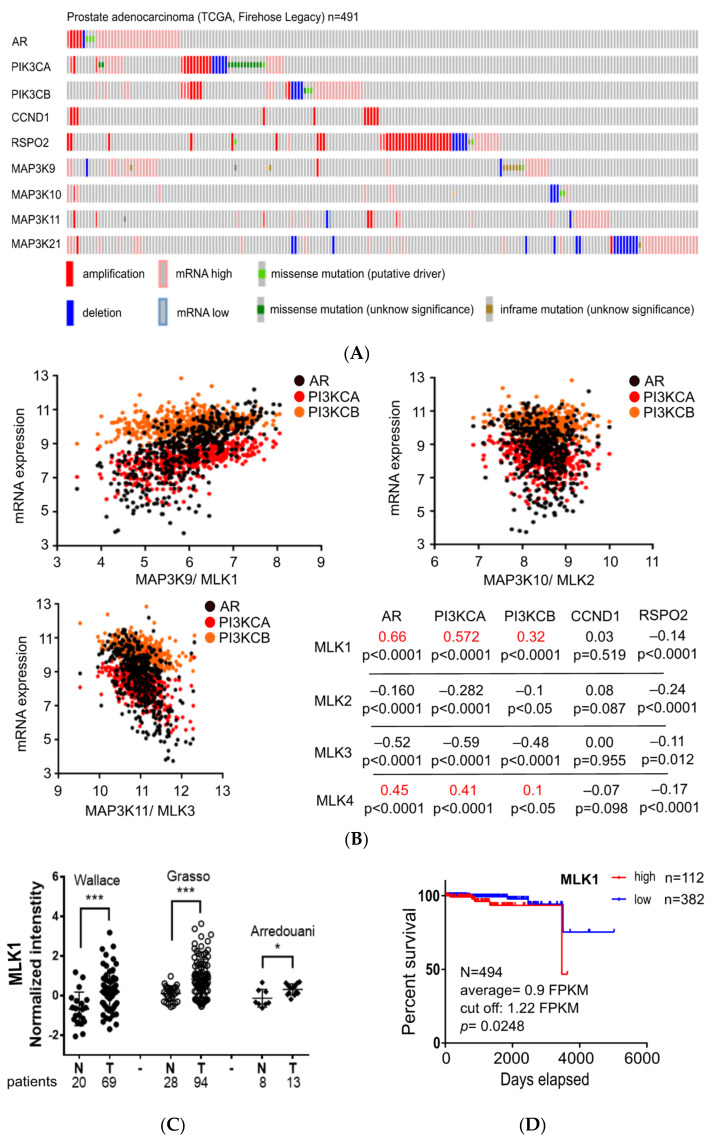
MLK1 mRNA expression is associated with prostate cancers. (**A**) Analyses of genetic alterations with a prostate cancer dataset (TCGA, Firehose Legacy) using the cBioPortal server. Each box represents a patient. (**B**) Correlation analysis for four MLK genes (MLK1-MLK4) with frequently-mutated genes (AR, PI3KCA, PI3KCB, CCND1, and RSPO2) identified in mCRPC. Correlation coefficients with positive values and statistical significance (*p* < 0.05) are labeled red. (**C**) Comparison of MLK1 mRNA expression in normal (N) and tumor (T) tissues using three clinical datasets (Wallace/GSE6956, Grasso/GSE35988, and Arredouani/Oncomine). (**D**) The impact of MLK1 mRNA expression on the survival of prostate cancer patients was analyzed in the Human Protein Atlas. FPKM: fragments per kilobase of transcript per million mapped reads. Student’s *t*-test: * *p* < 0.05, *** *p* < 0.001.

Since there are four genes in the MLK family (MLK1–MLK4), we decided to compare their associations with a signature gene profile of prostate cancers. We selected four frequently-amplified genes (AR, PIK3CA, PIK3CB, and CCND1) and one overexpressed gene (RSPO2) that were identified in tumors from metastatic CRPC (mCPRC) in a multi-institutional clinical sequencing study [25]. It was known that AR amplification sensitizes prostate cancers to low levels of androgen, that PI3K (PIK3CA/B) and Wnt (RSPO2) pathways can bypass the AR function for cell proliferation, and that CCND1 regulates the cell cycle; therefore, all five genes are involved in mCRPC [25]. Consistently, when we analyzed the genetic mutation profiles in cBioPortal [23], this showed that all the five genes exhibited gene amplification and increased mRNA levels (except CCND1) in a prostate cancer dataset (Firehose Legacy) from The Cancer Genome Atlas (TCGA) (Figure 1A).

We next performed correlation analyses of the MLKs with the five amplified genes. We found that both MLK1 and MLK4 behaved similarly, showing positive correlations with three of the five signature genes (AR, PI3KCA, and PI3KCB), while both MLK2 and MLK3 exhibited a negative correlation (Figure 1B). It should be noted that not all the MLKs showed a positive correlation with CCND1 and RSPO2, suggesting a functional independence. Since MLK1 had a better correlation with the signature genes than MLK4, we hypothesized that MLK1 is a tumor marker associated with prostate cancers. To further analyze the clinical significance, we examined the mRNA expression between normal and tumor samples using the Oncomine [21] and the GEO databases. As shown in Figure 1C, MLK1 mRNA expression was significantly increased in the prostate cancers compared with the normal tissues in several clinical datasets [17,18,19]. Furthermore, we asked whether MLK1 expression affects overall survival by analyzing the clinical data of TCGA using the Human Protein Atlas [24]. Indeed, the poor survival rate in the prostate cancers was associated with higher expression of MLK1 compared to the lower one (cut off = 1.22 FPKM, *p* = 0.0248, Figure 1D). In summary, our results suggest that MLK1 is a tumor-associated marker in prostate cancer.

### 3.2. Identification of an MLK1 Inhibitor, NSC14465

**Figure 2:**
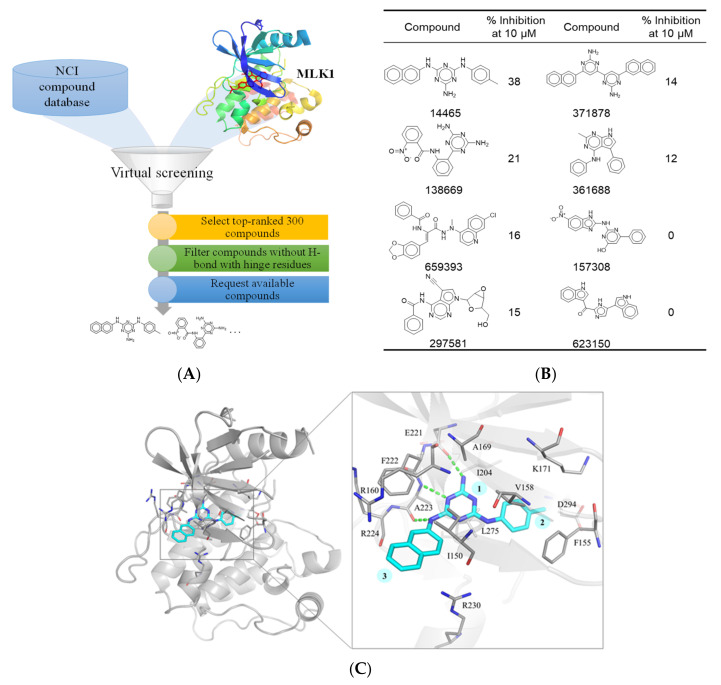
Selection of MLK1-targeting inhibitors. (**A**) Workflow of the structure-based virtual screening approach. The NCI compound library was virtually screened against the MLK1 structure (PDB ID: 3DTC). The resulting compounds were ranked by their MLK1 docking score (orange box). The top 300 compounds were selected and compounds with no hydrogen bonds were removed (green box). The remaining compounds were then selected (blue box) for in vitro kinase assay. (**B**) Structures and the inhibitory percentage of the selected compounds against MLK1. Compounds were tested by in vitro kinase assay using a concentration at 10 μM. (**C**) The docking pose of NSC14465. NSC4465 (blue) consists of a melamine (1) core with a toluene (2) and a naphthalene (3) ring in a meta position. Binding site residues (gray) are illustrated as lines and labeled as shown. Hydrogen bonds are illustrated as green dashes.

We next identified potential MLK1 inhibitors using a structure-based virtual screening approach. The workflow of in silico screening can be seen in Figure 2A. Compounds from the NCI compound database were docked using FlexX [14]. The compounds were then ranked based on their docking score, with the top-ranked 300 compounds selected for further filtering. Kinase inhibitors targeting the ATP binding site have a high preference for hydrogen bonds with hinge residues [16]. Therefore, docking poses that did not display a hydrogen bond to the MLK1 hinge residues (E221, F222, and A223) were removed. Compounds were visually inspected and selected based on availability. In total, eight compounds were selected for in vitro kinase analysis.

The selected compounds were tested at 10 µM. Of these, compound 14,465 (NSC14465) showed the greatest inhibition towards MLK1 (Figure 2B). To elucidate the protein–ligand interactions for inhibition, an interactive analysis of the docking pose was performed. Compound 14,465 contains a melamine core that forms one hydrogen bond and two hydrogen bonds to the backbones of hinge residues E221 and A223, respectively (Figure 2C). The melamine core also forms hydrophobic interactions with residues A169, A223, and L275. Two rings, a toluene and a naphthalene ring, are attached to the melamine amino groups in a meta position. The toluene moiety occupies a pocket to make hydrophobic interactions with residues F155, V158, and K171. The naphthalene ring yields an additional hydrophobic interaction to residue I150. These interactions suggest that NSC14465 can form favorable interactions within the MLK1 binding site. Together, this analysis suggests that NSC14465 is an MLK1 inhibitor.

### 3.3. Validation of Inhibitory Effects of NSC14465 on MLK1

**Figure 3:**
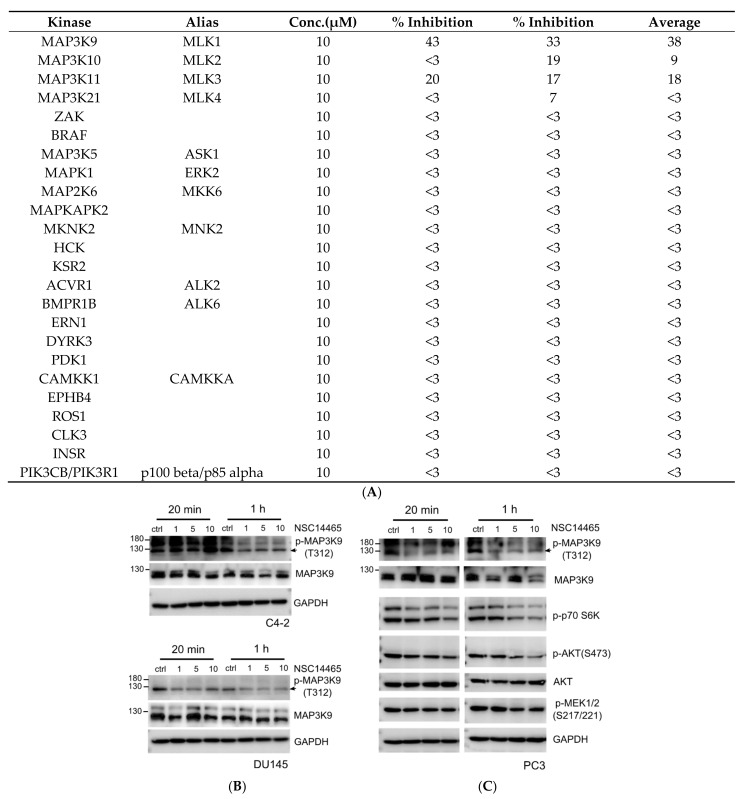
Validation of NSC14465 as a MLK1 kinase inhibitor. (**A**) Selectivity of the NSC14465. Five members in the MLK subgroup (MLK1-4, ZAK), two selected MAP3K (BRAF, MAP3K5), and seventeen other kinases were examined by in vitro kinase assay. The activity against each kinase was performed in duplicate and is shown as a percentage of inhibition (% inhibition). (**B**) In vivo suppression of MLK1 phosphorylation by NSC14465. Cells (C4-2 and DU145), treated with NSC14465 (1, 5, 10 μM) for different times (20 min and 1 h), were analyzed by Western blot assay with a phospho-specific antibody, p-MAP3K9 (T312), against MLK1. (**C**) In vivo suppression of the selected signaling in the PC3 cell line. PC3 cell lysates after treatment with NSC14465 (1, 5, 10 μM) for different times were analyzed by Western blot using the selected antibodies. An antibody against GAPDH was used as a loading control.

In order to validate that NSC14465 is a novel MLK1 inhibitor, we first analyzed its selectivity by kinase assay against a panel of kinases from different kinase families (24 kinases). As shown in Figure 3A, most kinases were not inhibited by NSC14465 (% inhibition ≤ 3), and NSC14465 preferentially inhibited the MLK1 (% inhibition~38) compared to other members in the MLK subgroup (% inhibition ≤ 18). Although we showed that both MLK1 and MLK4 had similar correlation patterns, with three frequently-amplified genes in mCRPC (Figure 1A), the two proteins exhibit structural difference, since NSC14465 only inhibited MLK1 but not MLK4. Therefore, these results further support that NSC14465 is a novel MLK1 inhibitor.

We next evaluated the inhibitory function of NSC14465 for MLK1 in cellular assays. Earlier studies showed that autophosphorylation at a threonine residue (Thr312) is crucial for the kinase activity of MLK1 [26]. Thr312 is located in the activation loop of the MLK1, and a threonine to alanine point mutation (T312A) will lead to reduced activity (1–2% of the wild type) [26]. As shown in Figure 3B, NSC14465 can suppress the MLK1 phosphorylation at Thr312 in two CRPC cell lines, C4-2 and DU145, in 1 h and 20 min, respectively; consistent with the inhibitory effects of MLK1 in the in vitro kinase assay. Earlier studies suggested that the ATK-S6K [27] and MEK-ERK [7] pathways were downstream of MLK1 signaling. Thus, we also demonstrated the inhibitory effects of NSC14465 in another CRPC cell line, PC3, and monitored the phosphorylation statuses in several kinases (Figure 3C). In addition to the phosphorylation of Thr312 in MLK1, the phosphorylation of AKT and S6K was also suppressed in 20 min, and reduced phosphorylation of MEK1/2 was apparent in 1 h (Figure 3C). When we asked whether the inhibitor affected the abundance of endogenous MLK1, there was a clear dosage-dependent reduction in C4-2 at 20 min; in addition, after a 1-h treatment with 10 μM dosage, the normalized MLK1 signal in PC3 was decreased by almost 30% (red, Supplementary Figure S1). It is possible that different cell lines show different kinetic and dosage responses to the inhibitor. In summary, we confirmed that NSC14465 inhibited MLK1 phosphorylation and its downstream signaling.

### 3.4. Characterization of the Anti-Proliferation and Anti-Migration Effects of NSC14465

**Figure 4:**
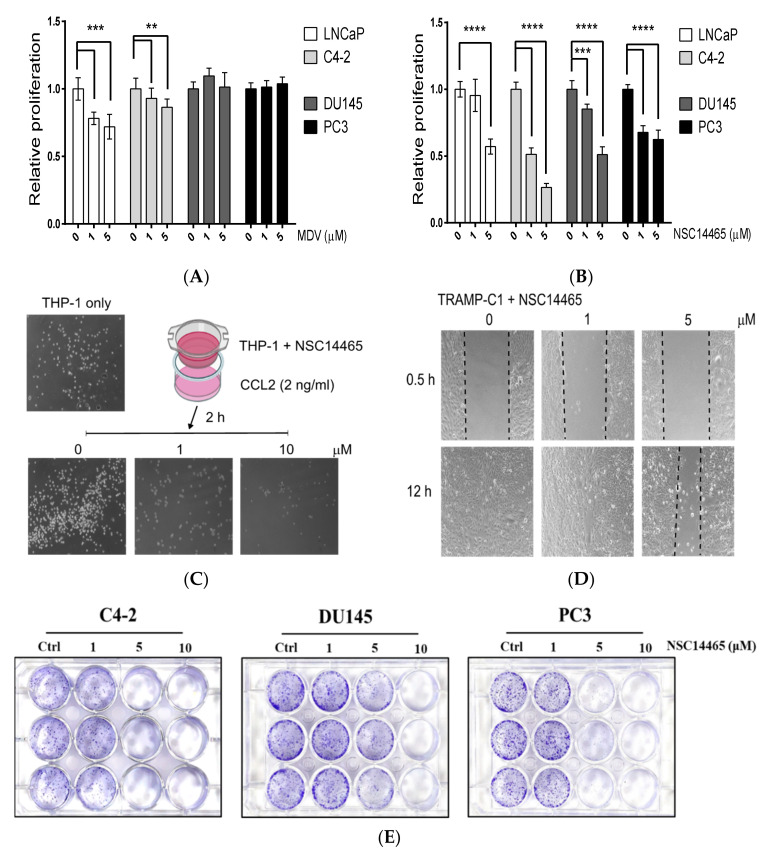
Anti-proliferation and anti-migration effects of NSC14465. (**A**) Monitoring proliferation of several human prostate cancer cell lines after treatment with an AR inhibitor, Enzalutamide (MDV), for 3 days. (**B**) Monitoring proliferation of several human prostate cancer cell lines after treatment with NSC14465 for 3 days. Student’s *t*-test: ** *p* < 0.01, *** *p* < 0.001, **** *p* < 0.0001. (**C**) Transwell analyses of a human monocyte cell line, THP-1, incubated with NSC14465 (0, 1, and 10 μM) in the upper chamber and the mouse CCL2 chemokine in the lower chamber for 2 h. (**D**) Migration analyses of a mouse cell line, TRAMP-C1, following NSC14465 treatment for 12 h. (**E**) Colony assays using human prostate cancer cell lines after treatment with NSC14465 for 5 days.

Since emerging evidence has suggested a role for MLK1 in malignant tumor progression [7,9,27], we examined the effects of NSC14465 in several prostate cancer cell lines. Compared to the AR-positive cell lines (LNCaP and C4-2), the AR-targeting drug, Enzalutamide, did not affect the proliferation of AR-negative cell lines (DU145 and PC3) (Figure 4A). However, NSC14465 exhibited anti-proliferation effects on, not only AR-positive and androgen-sensitive LNCaP, but also to all the CRPC cell lines (C4-2, DU145, and PC3) (Figure 4B). We also tested the anti-migration effect by monitoring the transwell activity of a human monocyte cell line, THP-1, in response to short time treatment with NSC14465. As shown in Figure 4C, NSC14465 can drastically suppress CCL2-promoted migration of THP-1. Similar results were observed using human prostate cancer (PC3) and mouse pancreatic cancer (6606PDA) cell lines (Supplementary Figure S2). Furthermore, we observed the anti-migration effects of NSC14465 using a wound healing assay in a mouse prostate cancer cell line, TRAMP-C1 (Figure 4D). In addition, we found that NSC14465 efficiently inhibited migration in human prostate cancer lines (PC3 and DU145) and one mouse pancreatic cancer cell line (6606PDA) (Supplementary Figure S3). Finally, we examined the anti-tumor activities of the compound by directly monitoring the colony formation abilities, an in vitro indication of tumor formation function. As shown in Figure 4E, the compound at 5 μM can drastically reduce the quantities of colonies from different CRPC cell lines. Again, NSC14466 exhibited similar anti-tumor abilities against both AR-positive (C4-2) and AR-negative (DU145 and PC3) cell lines in the same experimental setting. In summary, our results demonstrated that NSC14465 exhibits anti-proliferation and anti-migration effects, partly through inhibition of MLK1.

### 3.5. Demonstration of Anti-Tumor Effects of NSC14465 in a Syngeneic Pancreatic Cancer Model

**Figure 5:**
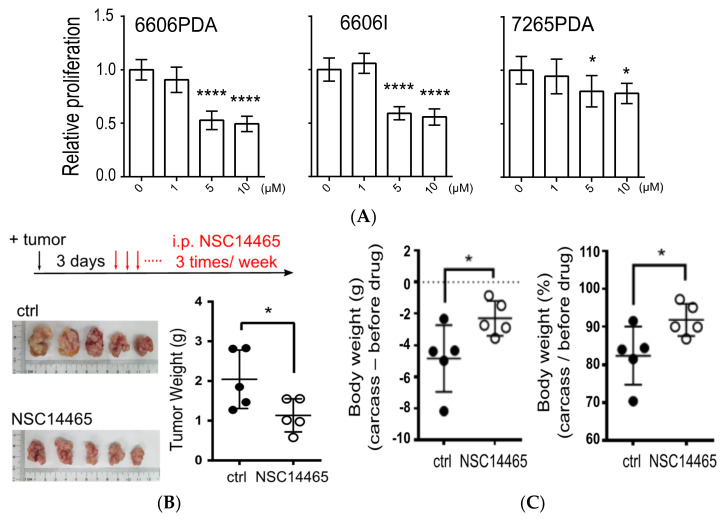
Anti-tumor effects of NSC14465. (**A**) Proliferation assays using mouse pancreatic cancer cell lines following treatment with NSC14465 for 3 days. (**B**) Comparison of tumor weights from mice orthotopically injected with 6606PDA followed by vehicle control or NSC14465 treatment. i.p. intraperitoneal injection. 1% DMSO/PBS (phosphate buffered saline) served as vehicle control. (**C**) Comparison of body weight changes in mice following NSC14465 treatment. Before drug: mice right before vehicle or NSC14465 treatments. Student’s *t*-test: * *p* < 0.05, **** *p* < 0.0001.

It was reported that reducing MLK1 expression can suppress MEK-ERK proliferation signaling and inhibit proliferation in pancreatic cancer cells [7]; therefore, development of MLK1 inhibitors could be a therapeutic means to treat pancreatic cancer. In order to examine the anti-tumor effects of NSC14465 in pancreatic cancers, we first performed a proliferation assay in three syngeneic mouse pancreatic cancer cell lines (6606PDA, 6606I, and 7265PDA) [10], and found that NSC14465 at dosages above 5 μM can inhibit proliferation (Figure 5A). We further asked whether NSC14465 exhibited anti-tumor effects in vivo using a syngeneic orthotopic pancreas carcinoma model [10]. From three days after injection of 6606PDA cells into the pancreas, mice were treated with NSC14465 three times a week until sacrifice (Figure 5B). About one month (27 days) after tumor injection, the mice were sacrificed due to apparent body weight loss in the vehicle control group. After we collected tumors and analyzed tumor weights, we demonstrated that NSC14465 reduced tumor size and tumor weight (Figure 5B).

The phenomenon of tumor-associated weight loss in mice recapitulated cancer cachexia in cancer patients [28]; thus, we further analyzed changes of body weight between the carcass and mice right before vehicle/or NSC14465 treatments (“before drug”, Figure 5C). Comparing to the vehicle treatment group, mice treated with NSC14465 had significantly reduced weight loss (Figure 5C). In another separate group, wild type male mice were treated only with NSC14465, without tumor injection, and no body weight loss was observed, suggesting the NSC14465 has no apparent toxic side effects in mice (Supplementary Figure S4). Our results supported that NSC14465 inhibited MLK1 and suppressed pancreatic cancers, preventing/or delaying development of cachexia.

## 4. Discussion

Although MLK1 is tightly correlated with AR expression in a prostate cancer clinical dataset (Figure 1A), we found that the inhibitory effects of the MLK1 inhibitor NSC14465 applied to, not only AR-positive, but also AR-negative, cell lines (Figure 4B). A possible explanation is that MLK1 is also critically involved in AR-negative prostate cancers. It is known that alternative pathways (e.g., Wnt and PI3K) are activated to support tumor cell proliferation during the development of the CRPC in response to AR inhibition [2]. The underlying mechanisms are partly due to a mutual inhibition between AR and those alternative pathways. For example, AR activation suppresses Wnt signaling [29], and inhibitors targeting PI3K signaling enhance expression of AR-targeted genes [30]. Therefore, alternative pathways may be de-repressed during AR inhibition and dominant in CRPC. Interestingly, we found that MLK1 not only had a strong correlation with AR signaling (Supplementary Figure S5A) but also with alternative pathways (i.e., PI3K and Wnt) (Figure S5B,C), suggesting that the MLK1 is also involved in some AR-suppressed processes that play major roles during AR inactivation. It is important to understand whether MLK1 is indeed involved in the development of CRPC when patients are treated with ADT. Thus, the development of an MLK1 inhibitor, either as a monotherapy or in combination with current AR-targeting agents, may provide a promising strategy for treating multiple types of prostate cancer.

Following the idea of targeting MLK1 as a therapeutic agent in treating prostate cancers, an earlier study in rats using a potent inhibitor of the MLK family showed that inhibiting MLKs did not affect either the survival or proliferation status of normal prostate epithelium in response to modulation of AR functions [31]. Therefore, targeting the MLK family may not elicit side effects in normal tissues, but may increase cytotoxic effects in the tumor cells of patients undergoing treatment with either ADT/or AR inhibitors. In addition, it is possible that the members of the MLK family participate in different types of CRPC. Two cell lines (C4-2B and 22Rv1) that express constitutively active AR variants (AR-V) are known to be resistant to a AR-targeting inhibitor, enzalutamide [32]; however, knockdown of MLK3 by a siRNA approach can sensitize both cell lines to the inhibitory effect of enzalutamide, albeit to different degrees (C4-2B: 73% vs. 22Rv1: 28%) [32]. Since NSC14465 exhibits comparable inhibitory effects against both the kinase activities of MLK1 (38% inhibition) and MLK3 (18% inhibition) (~twofold difference, Figure 3A), the anti-tumor effects of NSC14465 might depend on the relative abundance and activities of the MLK family members in tumor cells.

It is important to show anti-tumors effects in our syngeneic pancreatic cancer mouse model (Figure 5B). Although inhibition of MLK1 by siRNA was shown to facilitate anti-proliferation effects in vitro using pancreatic cell lines [7], it is not clear whether using small molecules targeting MLK1 can suppress pancreatic cancers in an animal model. Moreover, it was of significance that mice treated with NSC14465 did not exhibit tumor-associated weight loss (Figure 5C), considering that cancer cachexia has wide ranging effects in patients with pancreatic cancer (63–64%) [33]. One possible explanation to the prevention of cancer cachexia-like weight loss is that NSC14465 effectively suppresses pancreatic cancer cells in the microenvironment in the pancreas. Therefore, further modification of NSC14465 to improve its potency is a promising direction for treating pancreatic cancers and for improving quality of life. However, one might also interpret the lack of weight loss in the NSC14465 treatment group as an indication of a molecular model showing a direct involvement of MLK signaling in cancer cachexia. Consistently, it was shown that the MLK3/GDF15 axis serves an important role in inducing cancer cachexia [34]; thus, inhibition of MLKs may suppress the tumor-derived factors involved in cachexia. Based on our results, further investigation is required to clarify the connection between MLK1, or MLK3, and cachexia in pancreatic cancers.

## 5. Conclusions

The MLK1 is a relevant tumor marker in prostate cancer and targeting MLK1 can lead to anti-tumor effects in prostate and pancreatic cancers.

## Data Availability

MLK1 mRNA expression analysis by Oncomine (http://www.oncomine.org/, accessed on 2 June 2021) and Gene Expression Omnibus (http://ncbi.nlm.nih.gov/geo/, accessed on 2 June 2021). Correlation analysis by Gene Expression Profiling Interactive Analysis (GEPIA2) (http://gepia2.cancer-pku.cn/#index, accessed on 2 June 2021) and cBioPortal (http://www.cbioportal.org/, accessed on 2 June 2021). Survival analysis by The Human Protein Atlas (http://www.proteinatlas.org/, accessed on 2 June 2021).

## Supplementary Materials

The following are available online at https://www.mdpi.com/2079-7737/10/8/742/s1, Figure S1: Quantification of endogenous MLK1 and GAPDH. Figure S2: Transwell analyses of cancer cell lines. Figure S3: Wound healing assays of human prostate and mouse pancreatic cancer cell lines. Figure S4: Body weight changes in mice treated with NSC14465. Figure S5: Positive correlation of MLK1 (MAP3K9) with frequently-mutated genes identified in mCRPC. Full Western blot figures.

## References

[1] Siegel R.L., Miller K.D., Fuchs H.E., Jemal A. (2021). Cancer Statistics, 2021. CA Cancer J. Clin..

[2] Karantanos T., Corn P.G., Thompson T.C. (2013). Prostate cancer progression after androgen deprivation therapy: Mechanisms of castrate resistance and novel therapeutic approaches. Oncogene.

[3] Ferraldeschi R., Welti J., Luo J., Attard G., de Bono J.S. (2015). Targeting the androgen receptor pathway in castration-resistant prostate cancer: Progresses and prospects. Oncogene.

[4] Ritchie H. (2019). Cancer Death Rates Are Falling; Five-Year Survival Rates Are Rising. https://ourworldindata.org/cancer-death-rates-are-falling-five-year-survival-rates-are-rising.

[5] Neoptolemos J.P., Kleeff J., Michl P., Costello E., Greenhalf W., Palmer D.H. (2018). Therapeutic developments in pancreatic cancer: Current and future perspectives. Nat. Rev. Gastroenterol. Hepatol..

[6] Uhlik M.T., Abell A.N., Cuevas B.D., Nakamura K., Johnson G.L. (2004). Wiring diagrams of MAPK regulation by MEKK1, 2, and 3. Biochem. Cell Biol..

[7] Xia J., Cao T., Ma C., Shi Y., Sun Y., Wang Z.P., Ma J. (2018). miR-7 Suppresses Tumor Progression by Directly Targeting MAP3K9 in Pancreatic Cancer. Mol. Nucleic Acids.

[8] Cuevas B.D., Abell A.N., Johnson G.L. (2007). Role of mitogen-activated protein kinase kinase kinases in signal integration. Oncogene.

[9] Marusiak A.A., Edwards Z.C., Hugo W., Trotter E.W., Girotti M.R., Stephenson N.L., Kong X., Gartside M.G., Fawdar S., Hudson A. (2014). Mixed lineage kinases activate MEK independently of RAF to mediate resistance to RAF inhibitors. Nat. Commun..

[10] Zechner D., Burtin F., Amme J., Lindner T., Radecke T., Hadlich S., Kuhn J.P., Vollmar B. (2015). Characterization of novel carcinoma cell lines for the analysis of therapeutical strategies fighting pancreatic cancer. Cell Biosci..

[11] Baell J.B., Holloway G.A. (2010). New substructure filters for removal of pan assay interference compounds (PAINS) from screening libraries and for their exclusion in bioassays. J. Med. Chem..

[12] Lipinski C.A., Lombardo F., Dominy B.W., Feeney P.J. (2001). Experimental and computational approaches to estimate solubility and permeability in drug discovery and development settings. Adv. Drug Deliv. Rev..

[13] (2017). BIOVIA Pipeline Pilot, Release.

[14] Rarey M., Kramer B., Lengauer T., Klebe G. (1996). A fast flexible docking method using an incremental construction algorithm. J. Mol. Biol..

[15] Burley S.K., Bhikadiya C., Bi C., Bittrich S., Chen L., Crichlow G.V., Christie C.H., Dalenberg K., Di Costanzo L., Duarte J.M. (2021). RCSB Protein Data Bank: Powerful new tools for exploring 3D structures of biological macromolecules for basic and applied research and education in fundamental biology, biomedicine, biotechnology, bioengineering and energy sciences. Nucleic Acids Res..

[16] Xing L., Klug-Mcleod J., Rai B., Lunney E.A. (2015). Kinase hinge binding scaffolds and their hydrogen bond patterns. Bioorg. Med. Chem..

[17] Wallace T.A., Prueitt R.L., Yi M., Howe T.M., Gillespie J.W., Yfantis H.G., Stephens R.M., Caporaso N.E., Loffredo C.A., Ambs S. (2008). Tumor immunobiological differences in prostate cancer between African-American and European-American men. Cancer Res..

[18] Grasso C.S., Wu Y.M., Robinson D.R., Cao X., Dhanasekaran S.M., Khan A.P., Quist M.J., Jing X., Lonigro R.J., Brenner J.C. (2012). The mutational landscape of lethal castration-resistant prostate cancer. Nature.

[19] Arredouani M.S., Lu B., Bhasin M., Eljanne M., Yue W., Mosquera J.M., Bubley G.J., Li V., Rubin M.A., Libermann T.A. (2009). Identification of the transcription factor single-minded homologue 2 as a potential biomarker and immunotherapy target in prostate cancer. Clin. Cancer Res. Off. J. Am. Assoc. Cancer Res..

[20] Tomlins S.A., Mehra R., Rhodes D.R., Cao X., Wang L., Dhanasekaran S.M., Kalyana-Sundaram S., Wei J.T., Rubin M.A., Pienta K.J. (2007). Integrative molecular concept modeling of prostate cancer progression. Nat. Genet..

[21] Rhodes D.R., Kalyana-Sundaram S., Mahavisno V., Varambally R., Yu J., Briggs B.B., Barrette T.R., Anstet M.J., Kincead-Beal C., Kulkarni P. (2007). Oncomine 3.0: Genes, pathways, and networks in a collection of 18,000 cancer gene expression profiles. Neoplasia.

[22] Tang Z., Kang B., Li C., Chen T., Zhang Z. (2019). GEPIA2: An enhanced web server for large-scale expression profiling and interactive analysis. Nucleic Acids Res..

[23] Gao J., Aksoy B.A., Dogrusoz U., Dresdner G., Gross B., Sumer S.O., Sun Y., Jacobsen A., Sinha R., Larsson E. (2013). Integrative analysis of complex cancer genomics and clinical profiles using the cBioPortal. Sci. Signal..

[24] Uhlen M., Bjorling E., Agaton C., Szigyarto C.A., Amini B., Andersen E., Andersson A.C., Angelidou P., Asplund A., Asplund C. (2005). A human protein atlas for normal and cancer tissues based on antibody proteomics. Mol. Cell. Proteom..

[25] Robinson D., Van Allen E.M., Wu Y.M., Schultz N., Lonigro R.J., Mosquera J.M., Montgomery B., Taplin M.E., Pritchard C.C., Attard G. (2015). Integrative clinical genomics of advanced prostate cancer. Cell.

[26] Durkin J.T., Holskin B.P., Kopec K.K., Reed M.S., Spais C.M., Steffy B.M., Gessner G., Angeles T.S., Pohl J., Ator M.A. (2004). Phosphoregulation of mixed-lineage kinase 1 activity by multiple phosphorylation in the activation loop. Biochemistry.

[27] Liu Y., Hou J., Zhang M., Seleh-Zo E., Wang J., Cao B., An X. (2020). circ-016910 sponges miR-574-5p to regulate cell physiology and milk synthesis via MAPK and PI3K/AKT-mTOR pathways in GMECs. J. Cell Physiol..

[28] Argiles J.M., Busquets S., Stemmler B., Lopez-Soriano F.J. (2014). Cancer cachexia: Understanding the molecular basis. Nat. Rev. Cancer.

[29] Siu M.K., Chen W.Y., Tsai H.Y., Chen H.Y., Yin J.J., Chen C.L., Tsai Y.C., Liu Y.N. (2017). TCF7 is suppressed by the androgen receptor via microRNA-1-mediated downregulation and is involved in the development of resistance to androgen deprivation in prostate cancer. Prostate Cancer Prostatic Dis..

[30] Carver B.S., Chapinski C., Wongvipat J., Hieronymus H., Chen Y., Chandarlapaty S., Arora V.K., Le C., Koutcher J., Scher H. (2011). Reciprocal feedback regulation of PI3K and androgen receptor signaling in PTEN-deficient prostate cancer. Cancer Cell.

[31] Gao J., Isaacs J.T. (2001). Mixed lineage kinase (MLK) family members are not involved in androgen regulation of prostatic proliferation or apoptosis. Prostate.

[32] Kohrt S.E., Awadallah W.N., Phillips R.A., Case T.C., Jin R., Nanda J.S., Yu X., Clark P.E., Yi Y., Matusik R.J. (2021). Identification of Genes Required for Enzalutamide Resistance in Castration-Resistant Prostate Cancer Cells In Vitro. Mol. Cancer Ther..

[33] Kordes M., Larsson L., Engstrand L., Lohr J.M. (2021). Pancreatic cancer cachexia: Three dimensions of a complex syndrome. Br. J. Cancer.

[34] Lerner L., Tao J., Liu Q., Nicoletti R., Feng B., Krieger B., Mazsa E., Siddiquee Z., Wang R., Huang L. (2016). MAP3K11/GDF15 axis is a critical driver of cancer cachexia. J. Cachexia Sarcopenia Muscle.

