# Supplementary material for: Investigation of Anti-Tumor Effects of an MLK1 Inhibitor in Prostate and Pancreatic Cancers
Source: Biology (Basel). 2021 Aug 2;10(8):742. doi: 10.3390/biology10080742 (PMC8389662; doi:10.3390/biology10080742)

Original images for Western blotting

Article
Investigation of anti-tumor effects of an MLK1 inhibitor in prostate and pancreatic cancers

Yu-Ching Fan, Kai-Chen Hsu, Tony Eight Lin, Dietmar Zechner, Yuan-Chin Tsai

Gels in Figure 3B

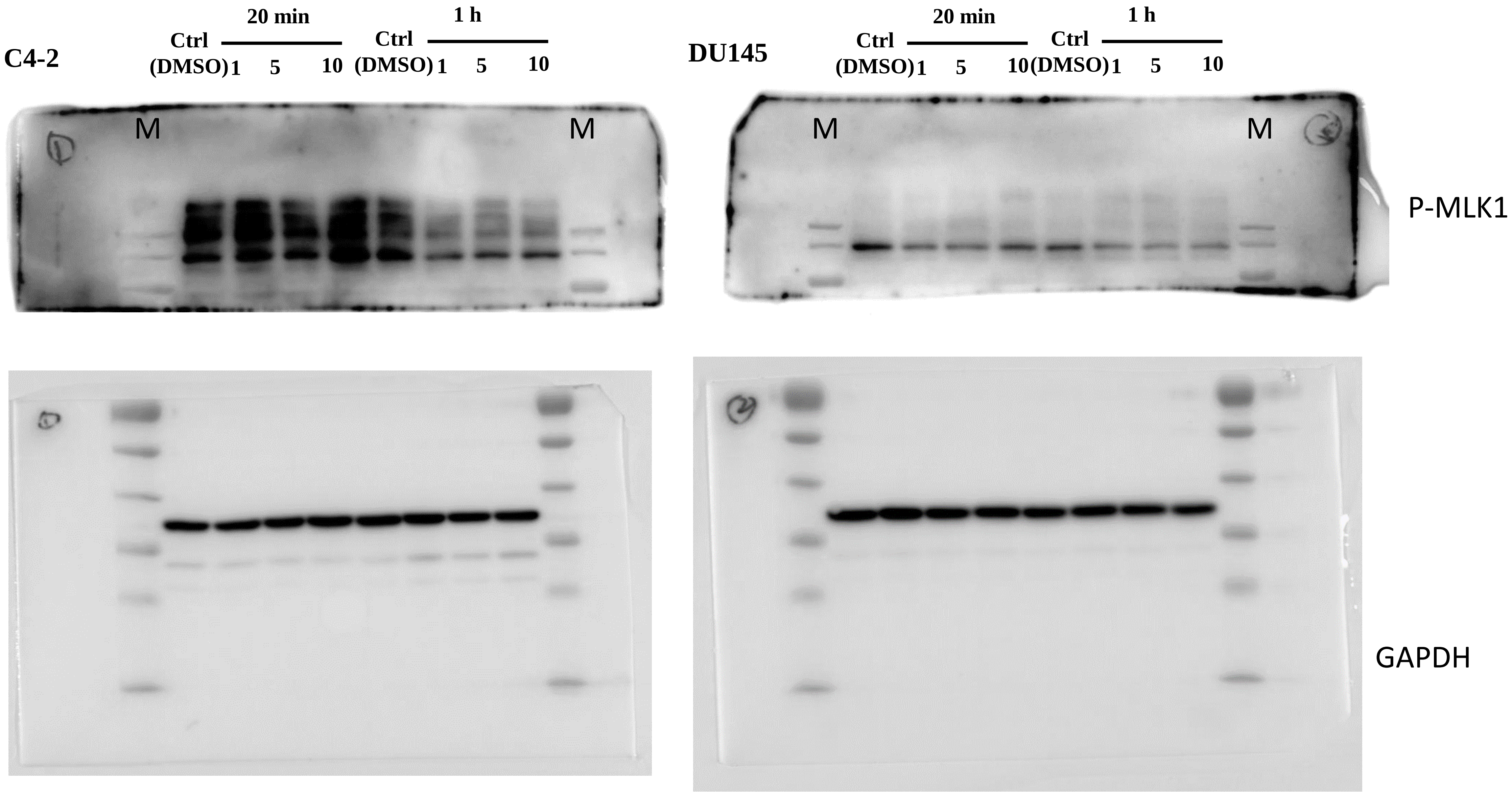

Gels in Figure 3C

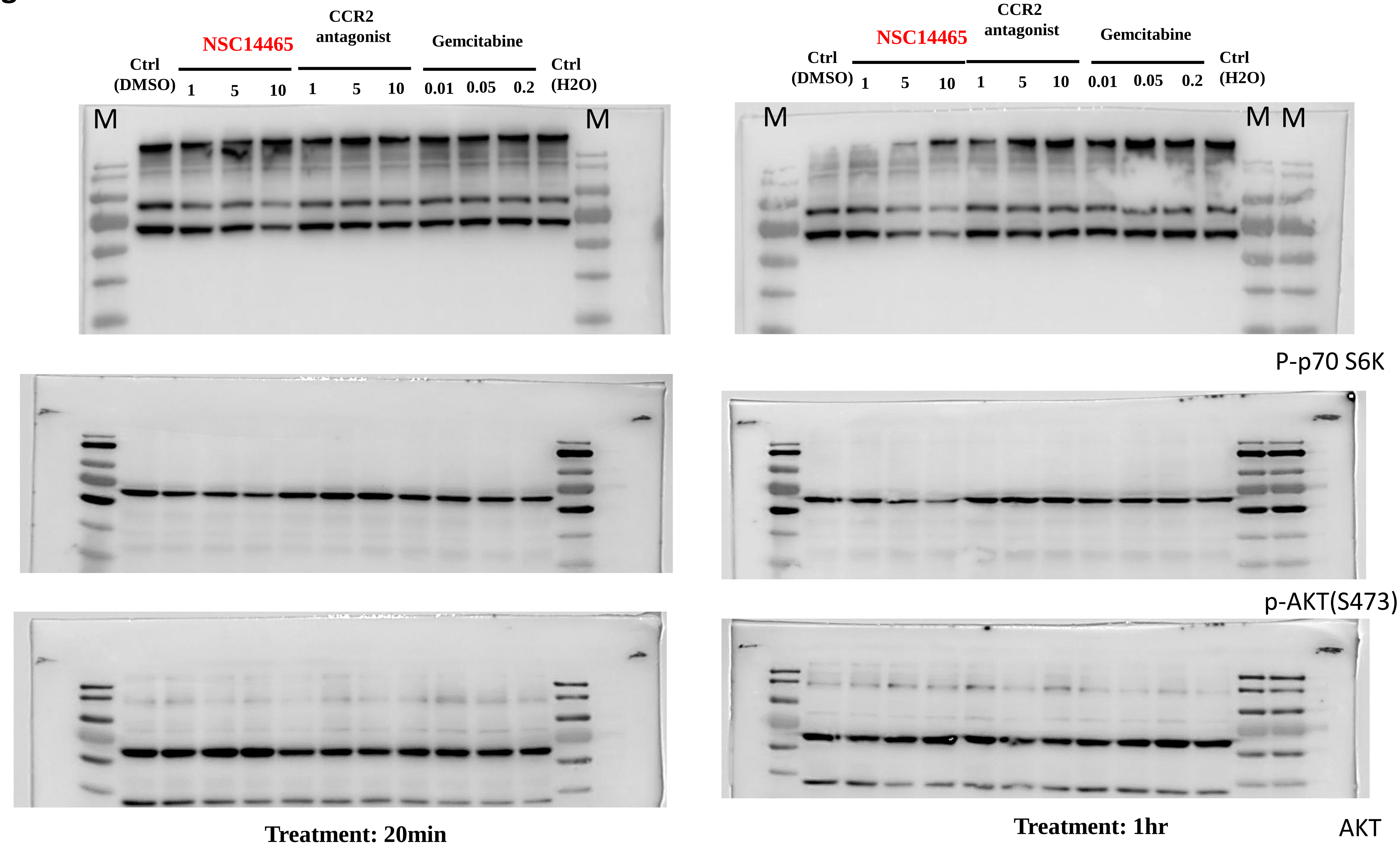

Gels in Figure 3C

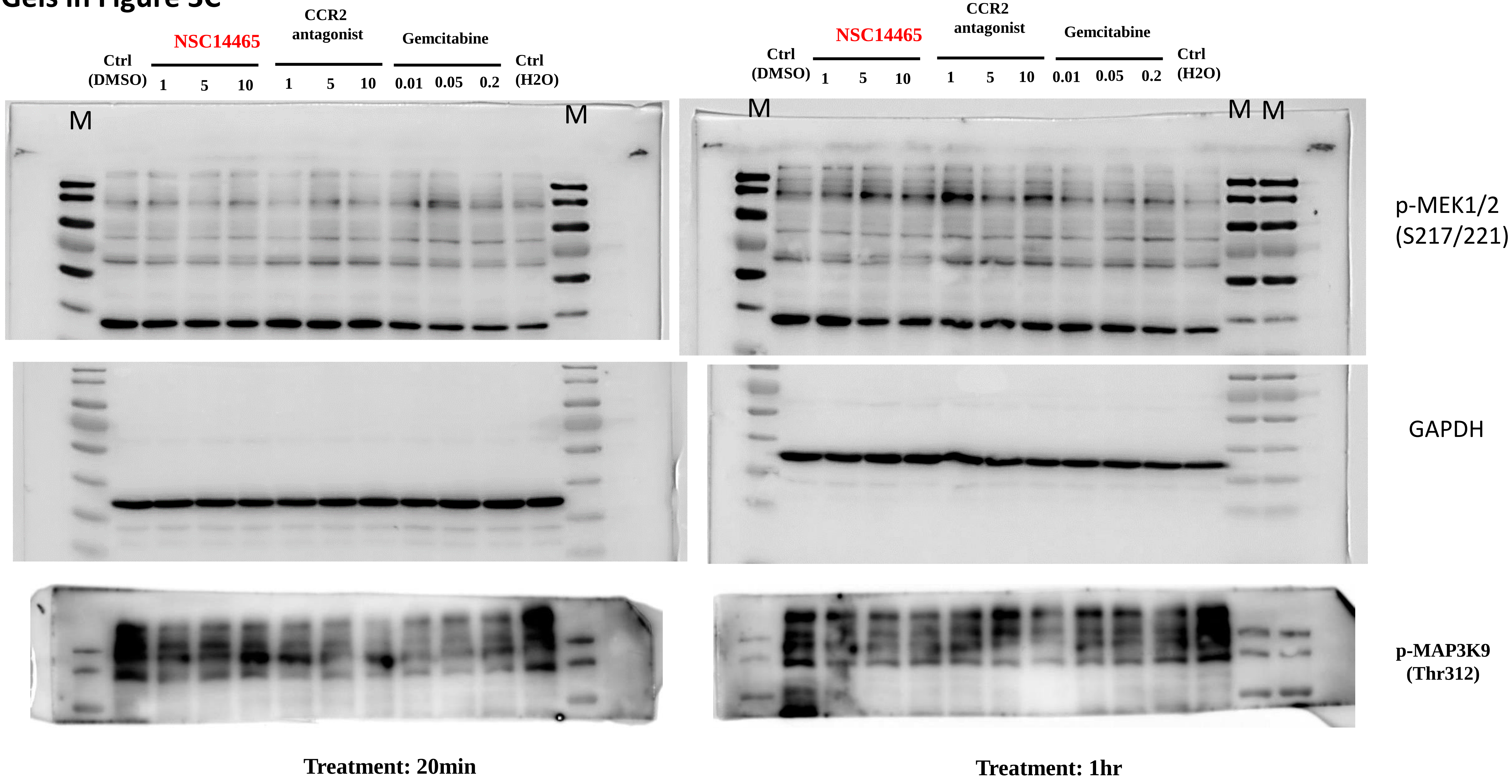

C4-2/
MAP3K9

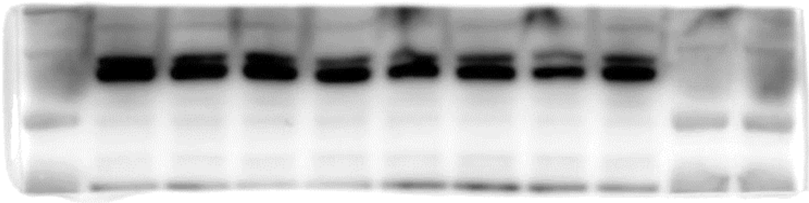

DU145/
MAP3K9

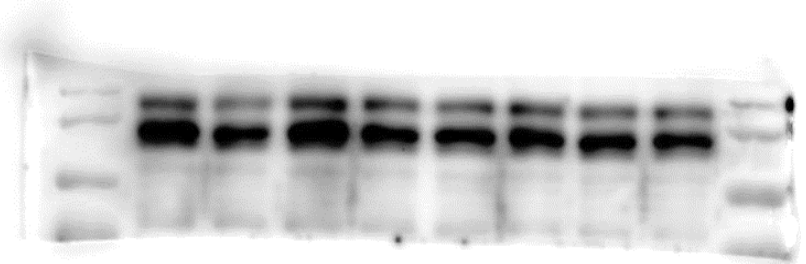

PC3/ MAP3K9

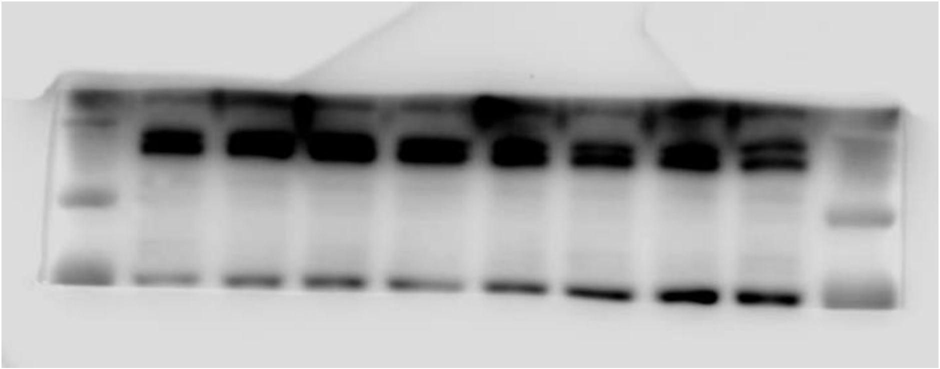

Supplement: Supplementary file 1 [file biology-10-00742-s001.zip › Full Western blot figures.pdf]
