# Supplementary material for: Investigation of Anti-Tumor Effects of an MLK1 Inhibitor in Prostate and Pancreatic Cancers
Source: Biology (Basel). 2021 Aug 2;10(8):742. doi: 10.3390/biology10080742 (PMC8389662; doi:10.3390/biology10080742)
Supplement: Supplementary file 1 [file biology-10-00742-s001.zip › Supplementary information.pdf]

Investigation of the anti-tumor effects of an MLK1 inhibitor in prostate and pancreatic cancers

Yu-Ching Fan, Kai-Chen Hsu, Tony Eight Lin, Dietmar Zechner, Yuan-Chin Tsai

Supplementary Figure S1
Quantification of endogenous MLK1 and the GAPDH

MLK1/GAPDH									
	20 min					1h			
μM	0	1	5	10		0	1	5	10
C4-2	100.0	88.2	82.1	68.8		100.0	117.3	93.3	135.8
DU145	100.0	77.9	104.7	93.4		100.0	95.6	97.3	99.1
PC3	100.0	94.6	108.6	102.9		100.0	103.6	97.9	71.9

Intensities of the immunoblotting signals in Figure 3B and 3C were quantified by ImageJ.

Supplementary Figure S2
Transwell analyses of cancer cell lines

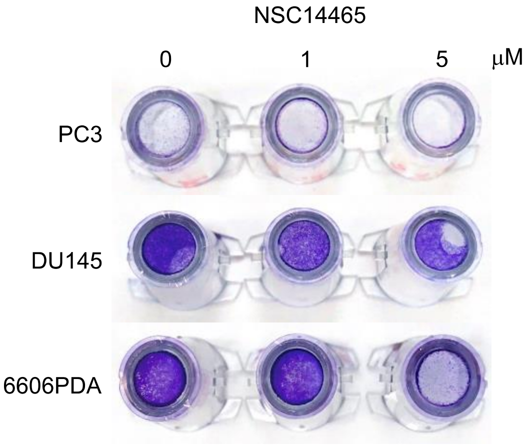

Transwell analyses using two human prostate cancer cell lines (PC3 and DU145), and one mouse pancreatic cancer cell line (6606PDA). Cells were incubated with NSC14465 (0, 1, and 5 μM) in the upper chamber. After 36 h, cells were stained with crystal violet.

Supplementary Figure S3
Wound healing assays of human prostate and mouse pancreatic cancer cell lines

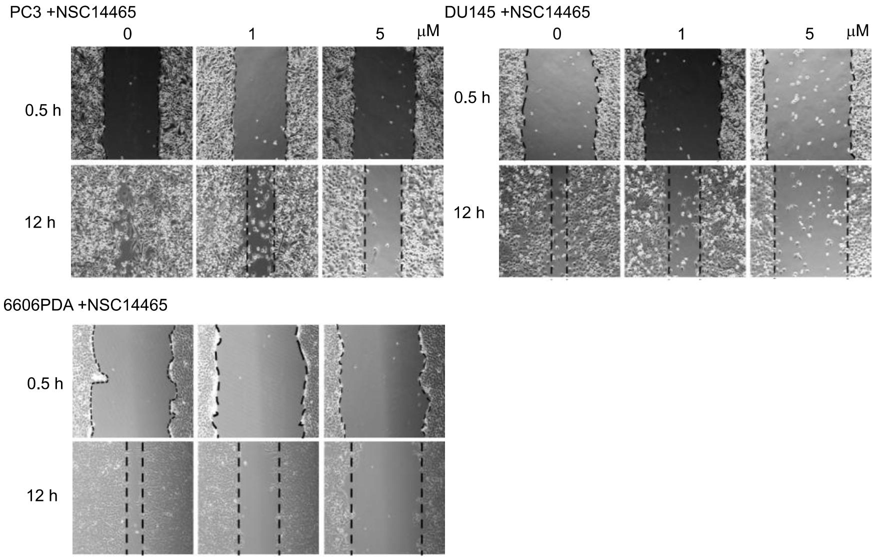

Migration analyses of two human prostate cancer cell lines (PC3 & DU145) and one mouse pancreatic cancer cell line (6606PDA) following NSC14465 treatment for 12 hours.

Supplementary Figure S4
Body weight changes in mice treated with NSC14465

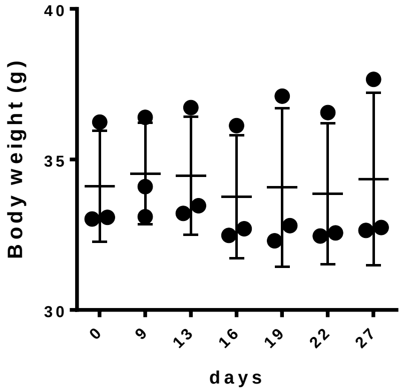

Without orthotopic injection of 6606PDA cells into the pancreas, 12-week-old male C57BL/6 mice were treated with NSC14465 three times a week until sacrifices. The body weights were monitored as shown.

Supplementary Figure S5
Positive correlation of MLK1 (MAP3K9) with frequently-mutated genes identified in mCRPC

A. MLK1 vs. AR-signaling (FOXA1, SPOP, and NCoR1)

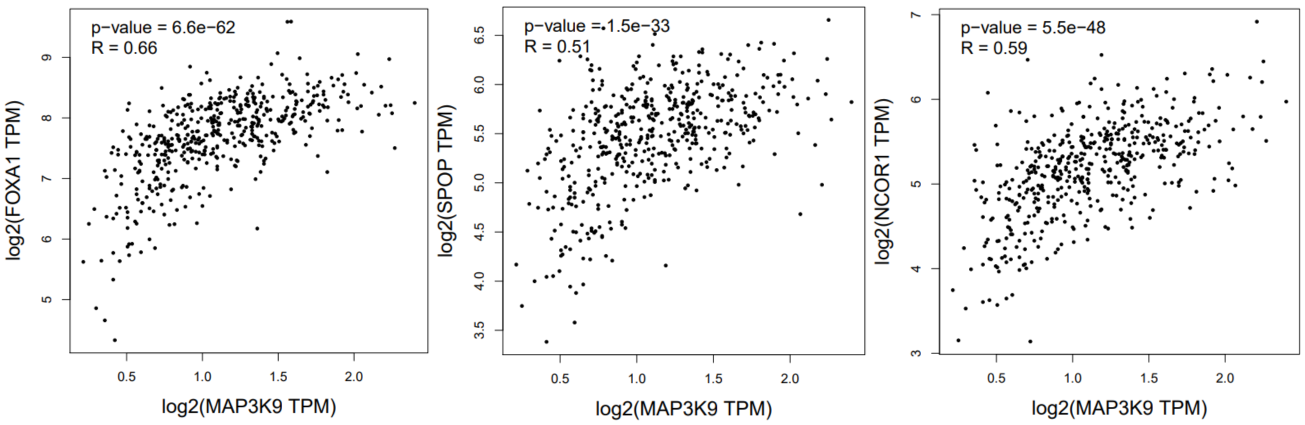

B. MLK1 vs. PI3K-signlaing (PIK3CA/B and AKT1)

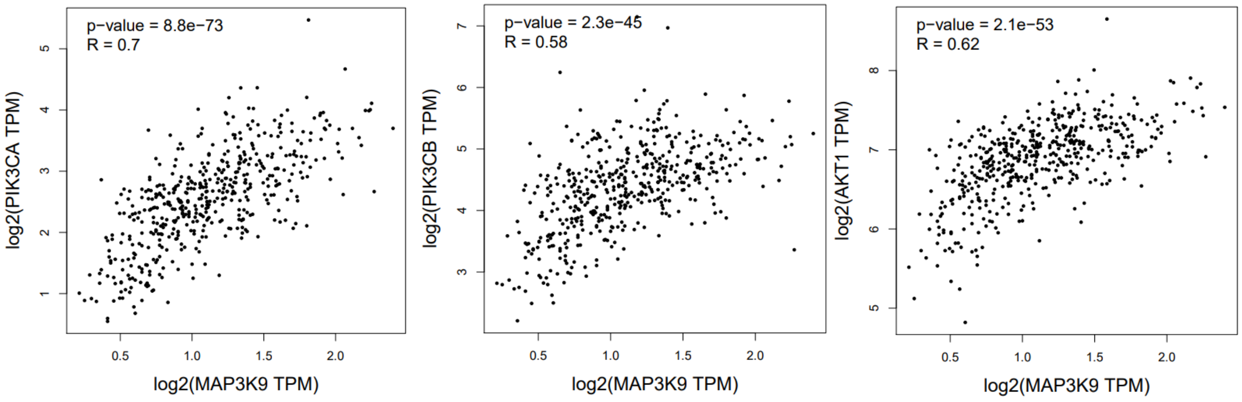

C. MLK1 vs. Wnt-signaling (APC and β-catenin)

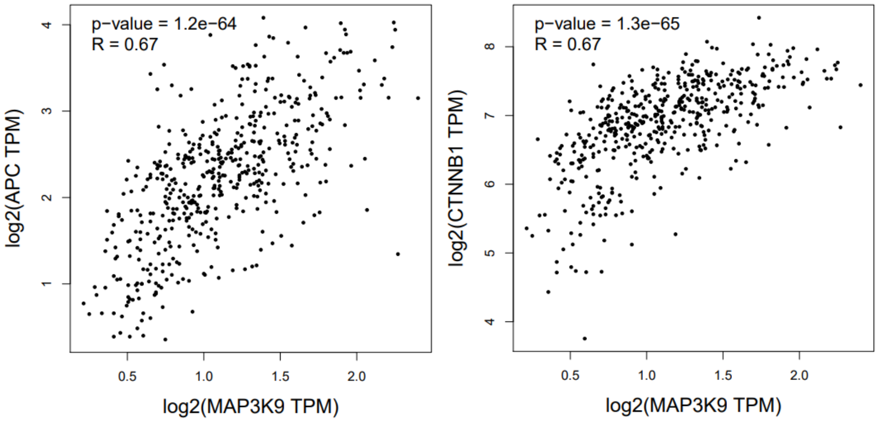

Correlation analyses between the MLK1 (MAP3K9) and biological pathways that were frequently mutated in metastatic CRPC [1]. The mRNA levels were analyzed by the GEPIA2 website. MAP3K9 is the gene name of MLK1. R: correlation coefficient using Spearman's rank correlation. TPM: transcripts per kilobase million. All the *p* values < 0.0001.

1. Robinson, D.; Van Allen, E.M.; Wu, Y.M.; Schultz, N.; Lonigro, R.J.; Mosquera, J.M.; Montgomery, B.; Taplin, M.E.; Pritchard, C.C.; Attard, G., et al. Integrative clinical genomics of advanced prostate cancer. *Cell* **2015**, *161*, 1215-1228, doi:10.1016/j.cell.2015.05.001.
